# A DFT Study on the Adsorption of H_2_S and SO_2_ on Ni Doped MoS_2_ Monolayer

**DOI:** 10.3390/nano8090646

**Published:** 2018-08-22

**Authors:** Huangli Wei, Yingang Gui, Jian Kang, Weibo Wang, Chao Tang

**Affiliations:** 1 College of Engineering and Technology, Southwest University, Chongqing 400715, China; hleighwei@sina.com (H.W.); tangchao_1981@163.com (C.T.); 2 State Grid Chongqing Shiqu Power Supply Company, Chongqing 400015, China; wind-k@163.com (J.K.); duduwowo@163.com (W.W.)

**Keywords:** SF_6_ decomposition components, Ni-MoS_2_ adsorbent, surface adsorption, DFT calculations

## Abstract

In this paper, a Ni-doped MoS_2_ monolayer (Ni-MoS_2_) has been proposed as a novel gas adsorbent to be used in SF_6_-insulated equipment. Based on the first-principles calculation, the adsorption properties of Ni-MoS_2_ to SO_2_ and H_2_S molecules, the main decomposition components of SF_6_ under a partial discharge (PD) condition have been studied. The adsorption energy, charge transfer, and structural parameters have been analyzed to find the most stable gas-adsorbed Ni-MoS_2_. Furthermore, the density of states (DOS), projected density of states (PDOS), and electron density difference were employed to explore the interaction mechanism between SO_2_, H_2_S, and the Ni-MoS_2_ surface. It is found that the H_2_S molecule and SO_2_ molecule interact with the Ni-MoS_2_ surface by strong adsorption energy. Therefore, we conclude that the interaction between these two kinds of gases and the Ni-MoS_2_ monolayer belongs to chemisorption, and the Ni-MoS_2_ monolayer might be a promising gas adsorbent for the fault recovery of SF_6_-insulated equipment. Additionally, we have to point out that all of the conclusions only considered the final adsorption energy, the barrier in the transition state has not been analyzed in this paper.

## 1. Introduction

Due to the excellent insulation and arc extinguishing properties of SF_6_, it has obtained a wide application in gas-insulated equipment, such as gas-insulated switchgear (GIS), gas-insulated breaker (GIB), and gas-insulated transformer (GIT). In addition, SF_6_-insulated equipment exhibits a great deal of advantages, such as a small occupied area requirement, little electromagnetic pollution, and high safety and reliability [1,2]. However, a certain amount of insulation defects inevitably occur in SF_6_-insulated equipment during the long-term running process, which may lead to partial discharge (PD) and the decomposition from SF_6_ to SF_x_ under the operating voltage [3,4]. Simultaneously, the SF_6_ gas-filled chamber inevitably contains trace amounts of impurities, such as H_2_O and O_2_ [5]. SF_x_ will quickly react with the H_2_O and O_2_ into various decomposition components (such as H_2_S, SO_2_, SOF_4_, SO_2_F_2_, SOF_2_, HF, CF_4_, and CO_2_, etc.) [1,6,7,8]. These decomposition components can significantly accelerate the corrosion and aging process of the insulation medium, resulting in insulation failure. In order to ensure the running stability of SF_6_-insulated equipment, the primary task is to maintain the purity of the filling gas, namely removing the decomposition components of SF_6_ [9,10]. Considering all of the SF_6_ decomposition components, H_2_S, SO_2_ gases, the main decomposition components under all types of PD conditions are the key removing target gases [11,12,13]. Thus, it is urgent to explore an effective adsorbent for H_2_S and SO_2_ removal.

Recently, along with the upsurge of research on graphene and other two-dimensional (2D) layered nanomaterials [14,15,16], the graphene-like MoS_2_ monolayer, exhibiting good chemical stability and thermal stability, high specific surface area, and high surface activity, has attracted much research interest for various applications, including electrochemical lithium storage, solid lubrication, catalysis, and gas adsorbents [17,18,19,20,21,22]. Among them, the application of MoS_2_ in gas adsorption has attracted much research attention in recent years. Liu et al. developed an ethanol gas sensor based on an indium oxide/molybdenum disulfide (In_2_O_3_/MoS_2_) nanocomposite, and investigated its gas-sensing properties to ethanol gas [23]. Dongwei Ma et al. improved the sensing properties of MoS_2_ to CO and NO by doping the MoS_2_ monolayer with Au, Pt, Pd, and Ni, concluding that introducing appropriate dopants could be a feasible method to improve the gas sensing performance of MoS_2_-based gas sensors [24]. The gas adsorption properties of common gases (CO, NO_2_, H_2_O, NH_3_) on a pristine monolayer MoS_2_ and a metal (V, Nb, Ta)-doped MoS_2_ monolayer reported by Jia Zhu et al., indicated that metal doping can significantly improve the adsorption properties, chemical activity, and sensitivity of the MoS_2_ monolayer [25].

On the one hand, metal atom doping provides a large number of free electrons, namely improving the electrical conductivity of the MoS_2_ monolayer. On the other hand, the strong orbital hybridization between the metal atom and gas molecules enhances the gas adsorption capacity of the MoS_2_ monolayer to gas molecules [24,25,26,27]. Herein, based on the first-principles calculation, we present an extensive theoretical investigation of the structure, total density of states (TDOS), and projected density of states (PDOS) of a Ni-doped MoS_2_ monolayer. Additionally, its gas adsorption performance towards the typical SF_6_ decomposition components H_2_S and SO_2_ has been systematically studied based on the analysis of structural parameters, TDOS, PDOS, and electron density difference. Furthermore, the gas adsorption mechanisms of the Ni-doped MoS_2_ monolayer to H_2_S and SO_2_ was obtained based on the research above. In order to ensure the practicability of the adsorbent, the adsorption property of the Ni-MoS_2_ monolayer towards SF_6_ molecule has also been studied. In conclusion, the Ni-doped MoS_2_ monolayer shows an ideal adsorption property to the target gases, signifying that it is a promising novel gas adsorbent used to ensure the running stability of SF_6_-insulated equipment.

## 2. Computational Details

All calculations were performed based on the density functional theory (DFT) [28,29]. The generalized gradient approximation (GGA) with the Perdew-Burke-Ernzerhof (PBE) was chosen to calculate the geometry optimization with the energy convergence accuracy, maximum stress, and max displacement set to 1 × 10^−5^ Ha, 2 × 10^−3^ Ha/Å, and 5 × 10^−3^ Ha, respectively [30,31]. The effect of spin-polarization was ignored in this paper, and Grimme dispersion correction has been introduced to describe the weak interactions, like van der Waals force. The double numerical plus polarization (DNP) was chosen as the basis set, the density functional semi-core pseudopotential (DSPP) was applied in core treatment, and the Monkhorst-pack k point mesh of 5 × 5 × 1 was employed [32,33]. The self-consistent (SCF) field tolerance was set to 1 × 10^−6^ Ha, and the DIIS size was set to 6 to speed up the convergence of SCF [34].

A 4 × 4 × 1 MoS_2_ monolayer supercell with a 20 Å vacuum slab, including 32 S atoms and 16 Mo atoms, was built in order to avoid the interaction between the adjacent cells. The optimized lattice constant of MoS_2_ is calculated to be 3.180 Å, which is in good agreement with other theoretical calculation results [35]. One Ni atom was placed on the top site of the Mo atom at the center of the 4 × 4 × 1 MoS_2_ monolayer supercell, bonding with three S atoms.

The adsorption energy (*E*_ads_) was calculated by Equation (1) [36]:

*E*_ads_ = *E*_Ni-MoS_2_/gas_ − *E*_Ni-MoS_2__ − *E*_gas_ (1)

where *E*_Ni-MoS_2_/gas_ is the total energy of the gas adsorbed Ni-MoS_2_, while *E*_Ni-MoS2_ and *E*_gas_ represent the total energy of the Ni-MoS_2_ monolayer and the total energy of free gas molecule, respectively. The more negative *E*_ads_ obtained after geometry optimization, the easier for the free gas molecule to be adsorbed on the Ni-MoS_2_ monolayer surface, indicating the adsorption system is more stable.

In addition, the charge transfer (*Q*_t_) between the gas molecule and Ni-MoS_2_ monolayer was calculated by Equation (2):

*Q*_t_ = *Q*_a_ − *Q*_b_ (2)

where *Q*_a_ and *Q*_b_ represent the amounts of carried charge of the gas molecules after and before gas adsorption, which were calculated by electron population analysis [37]. It is worth putting out that the value of *Q*_b_ is always 0 *e* in this paper. According to the definition, if *Q*_t_ is positive, the electrons transfer from gas molecule to the Ni-MoS_2_ monolayer. Additionally, the density of states (DOS) was calculated to analyze the interaction mechanism between gas molecules and the Ni-MoS_2_ monolayer [38].

## 3. Results and Discussion

### 3.1. Structures and Electronic Properties of H_2_S, SO_2_ and the Ni-MoS_2_ Monolayer

Firstly, the adsorption property of the Ni atom on the MoS_2_ monolayer was discussed according to the adsorption energy analysis and population analysis. The adsorption energy (*E*_ads_) of Ni atom on MoS_2_ monolayer was defined in the Equation (3):

*E*_ads_ = *E*_Ni-MoS_2__ − *E*_Ni_ − *E*_MoS_2__ (3)

The negative *E*_ads_ in Equation (3) indicates that the binding process is exothermic. As the most stable doping position of Ni on MoS_2_ monolayer is the top site of the Mo atom according to previous studies [24], therefore, only the structure of Ni-MoS_2_ with Ni doping on the top site of the Mo is discussed in this paper.

**Figure 1:**
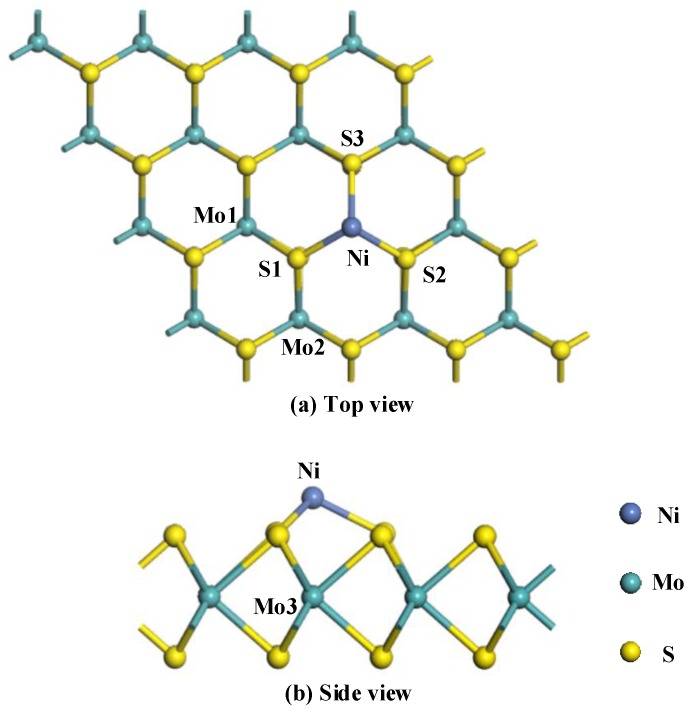
The structure of Ni-MoS_2_ monolayer: (**a**) top view; (**b**) side view.

As shown in Figure 1, The Ni atom above the Mo3 atom bonds with other three surrounding S atoms with a length of 2.121 Å, and there is no chemical bond between the doped Ni atom and the Mo3 atom because of the long distance between them (2.596 Å). The bond angle of the Mo1-S1-Mo2 near the Ni atom (81.3°) has slightly changed compared with that of the MoS_2_ monolayer without doping (82.0°), indicating that the doped Ni atoms have a strong interaction with the initial MoS_2_ monolayer structure, and the doping structure could be quite stable. Moreover, the large binding energy (3.495 eV) further confirmed the conclusion above. In addition, the charge transfer from the Ni atom to the MoS_2_ monolayer is 0.021 *e*.

**Figure 2:**
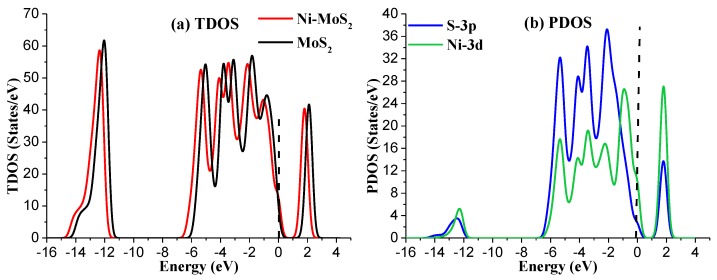
(**a**) The TDOS of Ni-MoS_2_; (**b**) the PDOS of Ni-MoS_2_, the dashed lines represent the Fermi level.

To further analyze the structural properties of Ni-MoS_2_ monolayer, the total density of states (TDOS) and projected density of states (PDOS) have been calculated as shown in Figure 2. For TDOS distribution, the electron distribution of the Ni-MoS_2_ monolayer around the Fermi level has slightly increased compared with that of the MoS_2_ monolayer, implying that the doping of the Ni atom has enhanced the metallic property of the MoS_2_ monolayer. As a result, the doped Ni atom acts as the active site for building interaction between the Ni-MoS_2_ monolayer and the target gas molecules. For PDOS distribution, the peaks of Ni-3d orbital and S-3p orbital overlap at −5.5 eV, −4.5 eV, −3.5 eV, −2.5 eV, and 2.0 eV, indicating that the S-3p orbital strongly hybridize with the Ni-3d orbital. Therefore, the Ni atom adsorbs on the surface of MoS_2_ monolayer by a stable structure.

**Figure 3:**
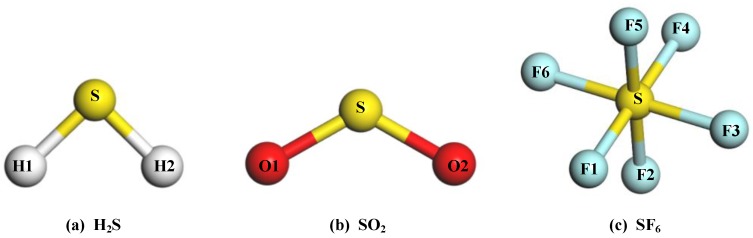
The molecular structures of gas molecules: (**a**) H_2_S; (**b**) SO_2_; (**c**) SF_6_.

The structures of the gas molecules are exhibited in Figure 3, and its specific structural parameters of the gas molecules are listed in Table 1. Additionally, the carried charge of S and O atom in the SO_2_ are 0.453 *e* and −0.227 *e*, respectively. The H atom in the H_2_S has a positive charge of 0.174 *e*, and the S atom has a negative charge of 0.348 *e*. For the SF_6_ molecule, the charge of the S atom is calculated to be 1.194 *e*, and the F atom is −0.199 *e*. These results are in agreement with the other theoretical calculation reports [39].

### 3.2. Adsorption of H_2_S Gas on the Ni-MoS_2_ Monolayer

**Figure 4:**
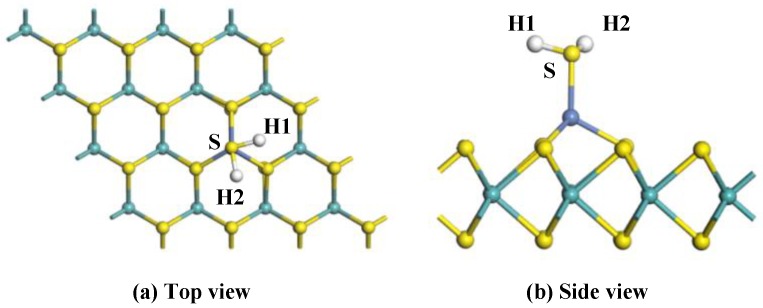
The configuration of the H2S-adsorbed Ni-MoS_2_ monolayer: (**a**) top view; (**b**) side view.

To analyze the adsorption properties of Ni-MoS_2_ monolayer to the target gas molecules, various initial approaching sites of H_2_S to the Ni-MoS_2_ monolayer were calculated in order to obtain the most stable adsorption structure. After optimization, only one typical adsorption structure was received, as shown in Figure 4 in the top view and side view, and its *E*_ads_, *Q*_t_, and specific structure parameters are shown in Table 2.

For adsorption system with structure shown in Figure 4a,b, a Ni-S bond with a length of 2.205 Å forms in the adsorption process, and the amount of the electrons transferred from the H_2_S molecule to Ni-MoS_2_ monolayer is up to 0.254 *e*, which means the S-Ni bond is not easy to break. However, the structure of the H_2_S has slightly changed after adsorption. The length of the H-S bond increases to 1.362 Å, the angle of the H1-S-H2 turned into 91.5°. The *E*_ads_ of H_2_S on the Ni-MoS_2_ monolayer is −1.319 eV, which is large enough to completely adsorb H_2_S. Though barrier exists in the transition state, but the change of the structure of H_2_S is not obvious, therefore, we conclude that the large *E*_ads_ can provide the energy to cross over the barrier. As a result, from the amount of electrons transfer and *E*_ads_, we conclude that the structure is the most stable structure for H_2_S adsorption.

**Figure 5:**
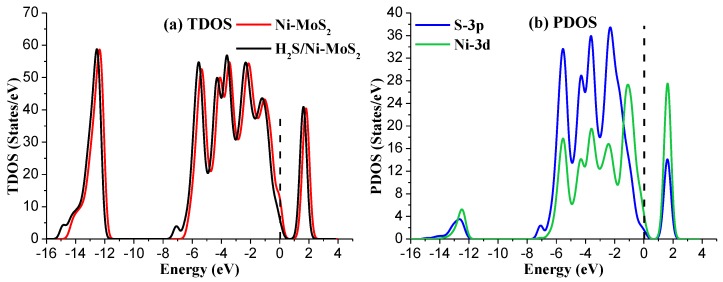
(**a**) The TDOS of Ni-MoS_2_ with and without H_2_S adsorption; (**b**) the PDOS of main interacted atoms, the dashed lines represent the Fermi level.

Figure 5 presents the TDOS and PDOS of Ni-MoS_2_ monolayer before and after H_2_S molecule adsorption. The TDOS after H_2_S molecule adsorption shows a significant increase near −7 eV and −15 eV. Due to the main contribution of the outer orbitals of interacted atom in the adsorption process, only the PDOS of the S-3p and Ni-3d was discussed. The PDOS analysis shows that the S-3p orbitals overlaps with the Ni-3d orbitals in the range from −6 eV to 0 eV, and the overlapped peaks of these two orbitals appear at about −5 eV, −4 eV, −3.5 eV, −2.5 eV, and 1 5 eV. The wide range of overlap fully verifies the strong hybridization between these two orbitals. The analysis of TDOS and PDOS furtherly confirms the strong interaction between H_2_S and Ni-MoS_2_ monolayer, and its structure is quite stable.

**Figure 6:**
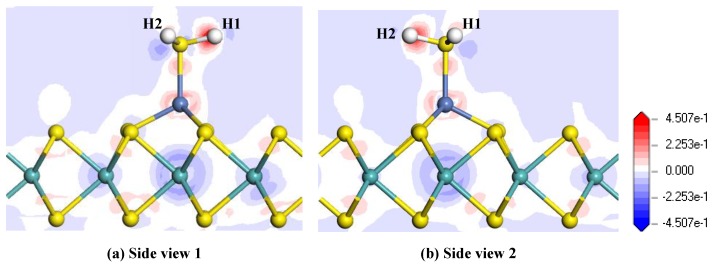
Electron density difference of the H_2_S-adsorbed Ni-MoS_2_ monolayer: (**a**) side view 1; (**b**) side view 2.

Figure 6 shows the electron density difference of the H_2_S adsorbed Ni-MoS_2_ monolayer from different side views, where the increase and decrease of the electron density are represented by the red and blue region, respectively. From the electron density difference, it is intuitive to analyze the charge distribution after the gas adsorption. It can be found that both of the H atoms receive electrons, while the electron density near the S atom and Ni atom suffer a reduction and increase, respectively, which is in agreement with the conclusion that the H_2_S molecule transfers quite a number of electrons to the monolayer. It is also interesting to notice that the electron density near the Mo atom below the Ni atom suffers an obvious reduction, and we assume that the electrons from the Mo atom made a contribution to the increase of the electron density surrounding H atoms. Therefore, the H_2_S molecule brings a dramatic change of electron density to the Ni-MoS_2_ monolayer.

In conclusion, considering the structure parameters, charge transfer, adsorption energy, DOS, and electron density difference of H_2_S adsorbed Ni-MoS_2_ monolayer, it is obvious that the interaction between the H_2_S and the Ni-MoS_2_ monolayer belongs to chemisorption. In consequence, this configuration is the most stable adsorption structure for H_2_S adsorption on a Ni-MoS_2_ monolayer. The Ni-MoS_2_ monolayer shows an outstanding adsorption property to H_2_S molecules.

### 3.3. Adsorption of SO_2_ Gas on a Ni-MoS_2_ Monolayer.

**Figure 7:**
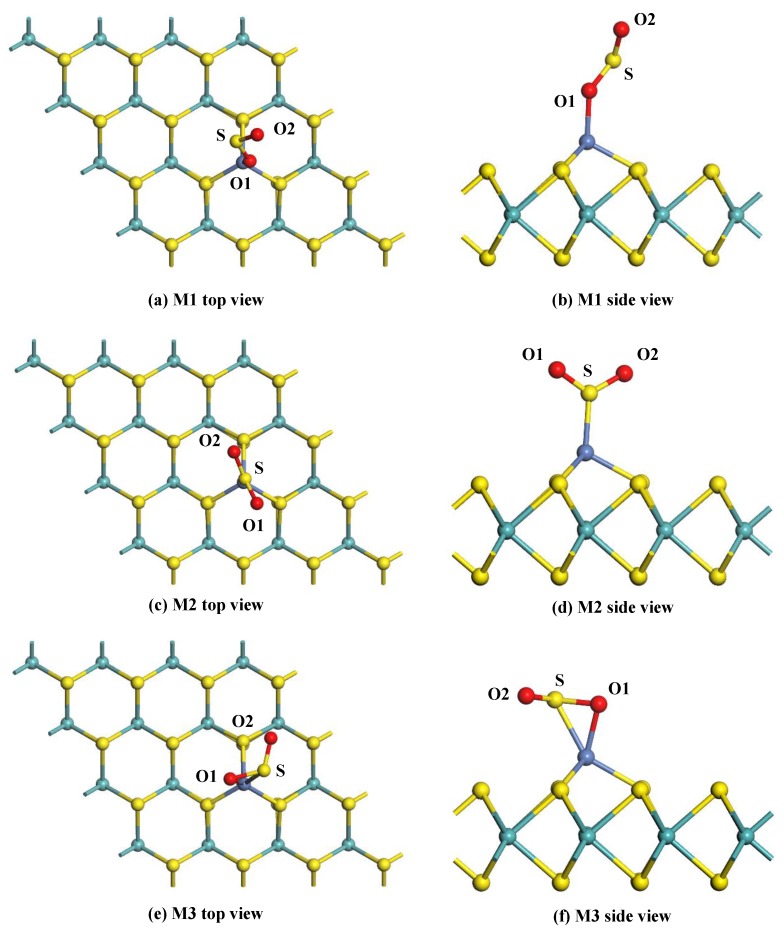
The adsorption configurations of the SO_2_-adsorbed Ni-MoS_2_ monolayer: (**a**,**b**) top and side view of M1; (**c**,**d**) top and side view of M2; and (**e**,**f**) top and side view of M3.

For the adsorption of SO_2_ gas, the gas molecule is initially placed at various sites to approach the surface of the Ni-MoS_2_ monolayer. Three typical adsorption structures were obtained after geometric optimization, as shown in Figure 7. Table 3 shows the structural parameters of these configurations.

Figure 7a,b shows the top view and side view of the M1 system. It can be seen that the SO_2_ molecule adsorbs on the Ni-MoS_2_ monolayer through the Ni-O1 bond, and the length of the Ni-O1 bond is 1.903 Å. The O1-S bond of SO_2_ adsorbed on the monolayer is 1.543 Å, which slightly longer than that of a free SO_2_ molecule (1.480 Å). The angle of the O1-S-O2 has decreased 5°. Thus, the structure of the SO_2_ molecule changes very little during the adsorption process. The *E*_ads_ of the M1 system is calculated to be −0.823 eV, 0.094 *e* transfers from the Ni-MoS_2_ monolayer to the SO_2_ molecule. Due to the strong interaction between SO_2_ and the Ni-MoS_2_ monolayer, the adsorption of the M1 system belongs to chemisorption.

The top view and side view of the M2 system are given in Figure 7c,d, the SO_2_ molecule adsorbs on the monolayer with a Ni-S bond length of 2.059 Å. From the structural parameters in Table 3, it is found that the structure of the SO_2_ molecule changes little after adsorption. The *E*_ads_ of the M2 system has increased to −1.382 eV compared to that in the M1 system, which manifests the stability of the M2 system. In Addition, only 0.016 *e* transfers from the SO_2_ molecule to the Ni-MoS_2_ monolayer in the M2 system, and the charge transfers from the S atom, O1 atom, and O2 atom are 0.050 *e*, −0.033 *e*, and −0.033 *e*, respectively.

The top view and side view of the M3 system are given in Figure 7e,f, the O1 atom and S atom are trapped by the Ni-MoS_2_ monolayer with bond lengths of 1.948 Å (Ni-O1) and 2.258 Å (Ni-S). Due to the strong interaction of the Ni-O1 bond and Ni-S bond, the *Q*_t_ of the M3 system is calculated to be −0.206 *e*, which is distinctly larger than that in the M1 and M2 system. From the structural parameters in Table 3, the bond distance and angle in the SO_2_ molecule insignificantly change after adsorption. The *E*_ads_ of M3 exhibited in Table 3 is −1.327 eV, which is slightly smaller than that in the M2 system.

Above all, according to the large amount of *E*_ads_ and charge transfer between the SO_2_ molecule and the M2 system, chemisorption of Ni-MoS_2_ monolayer to SO_2_ can be concluded. Although the *E*_ads_ of M3 system is very close to that of the M2 system, these two new built bonds between the SO_2_ molecule and Ni atom in M3 system means a higher barrier during the adsorption process. Therefore, the M2 system is the most stable configuration. To further verify the conclusion, the DOS and the electron density difference are intensively discussed below.

**Figure 8:**
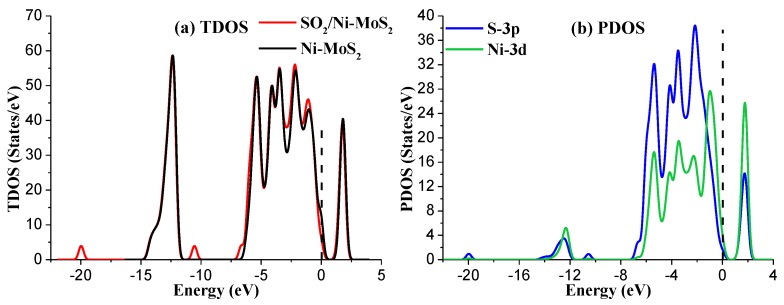
(**a**) The TDOS of Ni-MoS_2_ with and without SO_2_ adsorption; (**b**) the PDOS of the main interacted atoms in the M2 system. The dashed lines represent the Fermi level.

Figure 8a shows the TDOS of the M2 system. It is obvious that a small change occurs in TDOS around the area of −20 eV, −10.5 eV, −7 eV, −3 eV, and −1 eV for the SO_2_-adsorbed Ni-MoS_2_ monolayer. Similarly, as the adsorption process mainly contributed by of the outmost orbitals of atoms, only the PDOS of the S-3p and Ni-3d are discussed, as shown in Figure 8b. According to the PDOS results, the peaks of S-3p orbital and the Ni-3d orbital overlap at −5.5 eV, −4 eV, −2 eV, and 2 eV, suggesting that the interaction between SO_2_ and Ni-MoS_2_ monolayer is strong chemisorption, and its electronic structures are relatively active. Considering the large contribution of the S-3p orbital in the adsorption process, we confirm that the SO_2_ adsorption structure in the M2 system is very stable.

**Figure 9:**
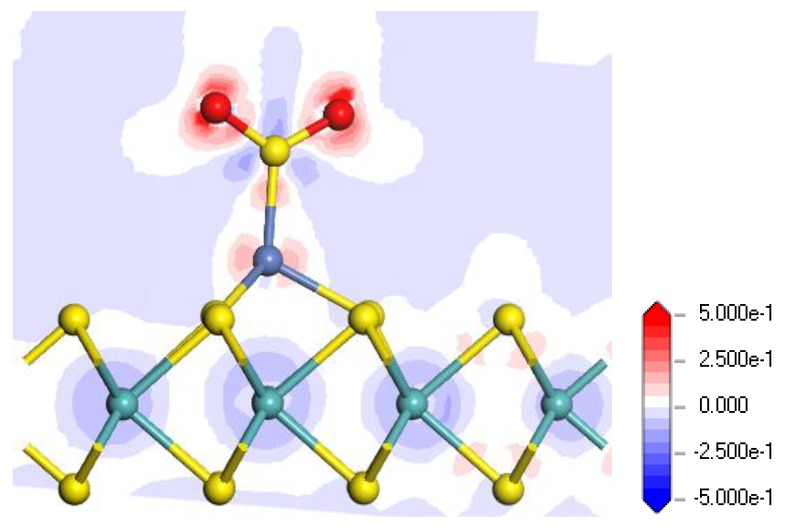
Electron density difference of the SO_2_-adsorbed Ni-MoS_2_ monolayer in the M2 system.

With respect to the electron density difference in the M2 system, shown in Figure 9, the increase and decrease of the electron density are represented by the red and blue regions, respectively. It is found that two O atoms in SO_2_ receive electrons, and the electron density near the S atom decreases during the adsorption. In generally, the SO_2_ molecule acts as an electron acceptor according to the electron density distribution.

### 3.4. Adsorption of SF_6_ Gas on the Ni-MoS_2_ Monolayer

**Figure 10:**
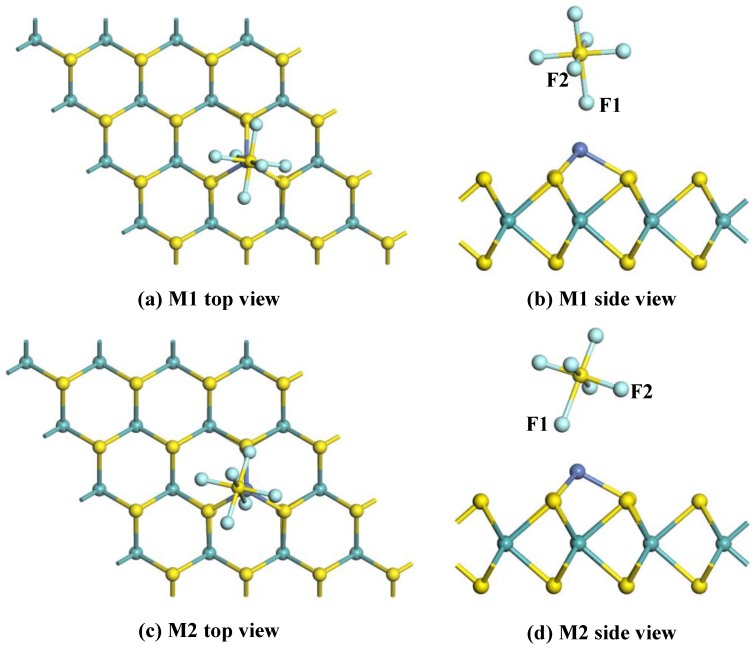
The adsorption configurations of the SF_6_ adsorbed Ni-MoS_2_ monolayer: (**a**,**b**) top and side view of M1; (**c**,**d**) top and side view of M2.

In order to ensure the practicability of the Ni-MoS_2_ adsorbent, the adsorption property of the Ni-MoS_2_ monolayer towards the SF_6_ molecule has also been studied, as SF_6_ will always be the largest part of components in SF_6_-insulated equipment. Various initial approaching sites of SF_6_ to the Ni-MoS_2_ monolayer were calculated in order to obtain the most stable adsorption structure. Two adsorption structures were received after geometric optimization, as shown in Figure 10 with different views. In addition, its adsorption energy, charge transfer, and other specific structural parameters are given in Table 4.

As the parameters show in the Table 4, the *E*_ads_ is only −0.174 eV for M1, and 0.181 eV for M2. *Q*_t_ is −0.445 *e* and −0.454 *e* for the M1 and M2 structures, respectively. Though the *d*_F1-S_ of SF_6_ suffers a very small increase compared with that of free SF_6_ molecule, it is still difficult to break its chemical bonds by the weak adsorption energy. Therefore, the SF_6_ molecule interacts with Ni-MoS_2_ monolayer by physisorption. Once H_2_S and SO_2_ decomposition components occur in SF_6_-insulated equipment, H_2_S and SO_2_ quickly fill the role of the adsorption of SF_6_ because of its strong adsorption energy of H_2_S and SO_2_. Additionally, the repulsion between gas molecules will block the interaction between the SF_6_ molecule and Ni-MoS_2_ monolayer. As a result, the Ni-MoS_2_ monolayer can be a good adsorbent to H_2_S and SO_2_ in a SF_6_ atmosphere.

## 4. Conclusions

In this study, a Ni-MoS_2_ monolayer material has been proposed as a potential adsorbent to remove the typical decomposition components of SF_6_ under partial electric discharge: H_2_S and SO_2_. All of the calculations performed with respect to density functional theory analysis and all of the conclusions only considered the final adsorption energy; the barrier in transition state has not been analyzed in this paper. Various adsorption models of H_2_S and SO_2_ molecules on the Ni-MoS_2_ monolayer were built to find the most stable adsorption structure by analyzing the adsorption energy, charge transfer, and other structural parameters. To further analyze the interaction mechanism, the DOS, PDOS and electron density difference were presented and analyzed. We concluded that H_2_S and SO_2_ tend to adsorb on the surface of Ni-MoS_2_ monolayer by chemisorption, and the adsorption energy of the H_2_S and SO_2_ is up to −1.319 eV and −1.382 eV, respectively, indicating that the interaction between these two kinds of gases and the Ni-MoS_2_ monolayer is pretty strong. Additionally, the weak physisorption between SF_6_ and the Ni-MoS_2_ monolayer provides the basis for selectively adsorbing H_2_S and SO_2_ from the SF_6_ atmosphere. Therefore, the Ni-MoS_2_ monolayer might be a promising gas adsorbent to remove these two typical decomposition components of SF_6_, which plays a key role in enhancing the running stability of SF_6_-insulated equipment.

## Figures and Tables

**Table 1:** The structural parameters of H_2_S, SO_2_, and SF_6_.

Gas Molecule	Bond Angle (°)		Bond Length (Å)	
	Type	Angle	Type	Length
SO_2_	O1-S-O2	120.2	O1-S	1.480
H_2_S	H1-S-H2	91.2	H1-S	1.356
SF_6_	F1-S-F2	90.0	F1-S	1.616

**Table 2:** The *E*_ads_, *Q*_t_ and structural parameters of the H_2_S-adsorbed Ni-MoS_2_ monolayer.

Configuration	*E*_ads_ (eV)	*Q*_t_ (*e*)	*d*_H1-S_ (Å)	*d*_Ni-H2S_ (Å)	∠*H*1-*S*-*H*2 (°)
Figure 4	−1.319	0.254	1.362	2.205	91.5

**Table 3:** The structural parameters of adsorption configurations of the SO_2_-adsorbed Ni-MoS_2_ monolayer.

Configuration	*E*_ads_ (eV)	*Q*_t_ (*e*)	*d*_O1-S_ (Å)	*d*_O2-S_ (Å)	*d*_Ni-S_ (Å)	*d*_Ni-O1_ (Å)	∠O1-S-O2 (°)
M1	−0.823	−0.094	1.543	1.489	-	1.903	115.2
M2	−1.382	−0.016	1.481	1.481	2.059	-	119.2
M3	−1.327	−0.206	1.575	1.494	2.258	1.948	116.6

**Table 4:** The structural parameters of adsorption configurations of the SF_6_ adsorbed Ni-MoS_2_ monolayer.

Configuration	*E*_ads_ (eV)	*Q*_t_ (*e*)	*d*_Ni-F1_ (Å)	*d*_Ni-F2_ (Å)	*d*_F1-S_ (Å)	*d*_F2-S_ (Å)	∠F1-S-F2 (°)
M1	−0.174	−0.445	1.875	3.560	1.796	1.684	89.3
M2	−0.181	−0.454	1.871	3.511	1.851	1.685	89.0
